# Identification and mechanistic analysis of neurovascular coupling related biomarkers for diabetic macular edema

**DOI:** 10.3389/fmolb.2024.1332842

**Published:** 2024-09-13

**Authors:** Tianpeng Chen, Shufan Sheng, Jing Chen, Xiaole Wang, Yanxing Shang, Chengwei Duan, Caixia Liang, Yu Song, Dongmei Zhang

**Affiliations:** ^1^ Medical Research Center, Affiliated Hospital 2 of Nantong University, Nantong, China; ^2^ Jiangsu Provincial Medical Key Discipline (Laboratory) Cultivation Unit, Medical Research Center, Nantong First People’s Hospital, Nantong, China; ^3^ Nantong Municipal Medical Key Laboratory of Molecular Immunology, Medical Research Center, Nantong First People’s Hospital, Nantong, China; ^4^ Nantong Municipal Key Laboratory of Metabolic Immunology and Disease Microenvironment, Medical Research Center, Nantong First People’s Hospital, Nantong, China; ^5^ Department of Ophthalmology, Affiliated Hospital 2 of Nantong University, Nantong, China

**Keywords:** diabetic macular edema, diabetic retinopathy, neurodegeneration, neurovascular coupling, bioinformatic analysis

## Abstract

**Introduction:**

Diabetic macular edema (DME) is a major cause of vision loss in the sick with diabetic retinopathy. The occurrence of DME is closely related to the breakdown of neurovascular coupling; however, its underlying mechanism has not been fully elucidated. The aim of this study was to investigate the diagnostic biomarkers and potential molecular mechanisms associated with neurovascular coupling in DME.

**Methods:**

The differential expression analysis, STEM, and WGCNA were performed from GSE160306 to identify hub genes. The gene expression was validated by RT-qPCR. The relevant mechanisms of action were investigated through GO, KEGG, and GSEA analyses, as well as co-expression networks. Additionally, the LASSO regression analysis and a nomogram were used to demonstrate the diagnostic effectiveness of the model. Finally, the GenDoma platform was utilized to identify drugs with potential therapeutic effects on DME.

**Results:**

Neurotrophic factor receptor (NGFR) was identified as a hub gene related to neurovascular coupling and DME. The expression of NGFR was verified by RT-qPCR in *vitro* cells. GSEA analysis indicated that high expression of NGFR may affect immunity and inflammatory pathway, thereby regulating neurovascular coupling and mediating the development of DME. The NGFR co-expression network was constructed, which exhibited the correlation with the neurotrophin signaling pathway. Moreover, a diagnostic model for DME based on NGFR and PREX1 demonstrated relatively good diagnostic performance using LASSO regression analysis and the nomogram. And then the GenDoma platform identified drugs with potential therapeutic effects on DME.

**Conclusion:**

The high expression of NGFR may lead to abnormal neurovascular coupling and participate in the occurrence of DME by regulating the immunity, inflammatory and neurotrophin signaling pathway. Detection of NGFR and related expression genes may be beneficial for monitoring the occurrence and development of DME.

## Introduction

Diabetes is a global chronic disease, the incidence of which is rising annually and showing a trend among younger people. It is estimated that by 2030, 191 million people aged 26 to 75 will be blinded by diabetes, posing a substantial socioeconomic burden on the healthcare system (Ai et al., 2020; Ding and Wong, 2012). Hyperglycemia, dyslipidemia, insulin resistance, and dysregulation of metabolic pathways are considered to be the core pathophysiological mechanisms of diabetes, leaing to a series of complications involving multiple organ functions (Aldosari et al., 2022; Qin and Zou, 2022). Diabetic macular edema (DME) is a major cause of vision loss in diabetic patients, with its incidence increasing alongside the progression of the disease and stages of retinopathy (Starace et al., 2021; Moisseiev and Loewenstein, 2017). DME can occur at any stage of diabetic retinopathy (DR), affecting approximately 30% of adults with diabetes for 20 years or more, and about 71% of adults with proliferative diabetic retinopathy (PDR) (Kim et al., 2019; Miller and Fortun, 2018). Therefore, in order to carry out effective clinical diagnosis and treatment, there is a pressing need to identify biomarkers/target that can easily and accurately screen for DME (Rhee et al., 2021).

The pathogenesis of DME is multifactorial, with increased oxidative stress, vascular dysfunction, inflammation, and neurodegeneration playing significant roles (Kuroiwa et al., 2021). Neurovascular coupling is necessary for the retinal vasculature to adapt to changes in neurological function and increased metabolic demand, where active neurons induce local vasodilation in the neural tissue, increasing blood flow, providing sufficient energy and rapid removal of metabolic waste (Kaplan et al., 2020; Garhofer et al., 2020). Studies have shown that one of the most significant functional changes in patients with diabetes is the loss of vascular regulation due to changes in neural activity, and this retinal neurovascular coupling impairment often occurs early in diabetes (Moran et al., 2016; Fletcher et al., 2022). However, the response of vascular regulation to neural activity in neurovascular coupling dysfunction is complex. The molecular mechanism and potential biomarkers or targets of neurovascular coupling injury leading to the development of DME remain to be further studied.

**FIGURE 1:**
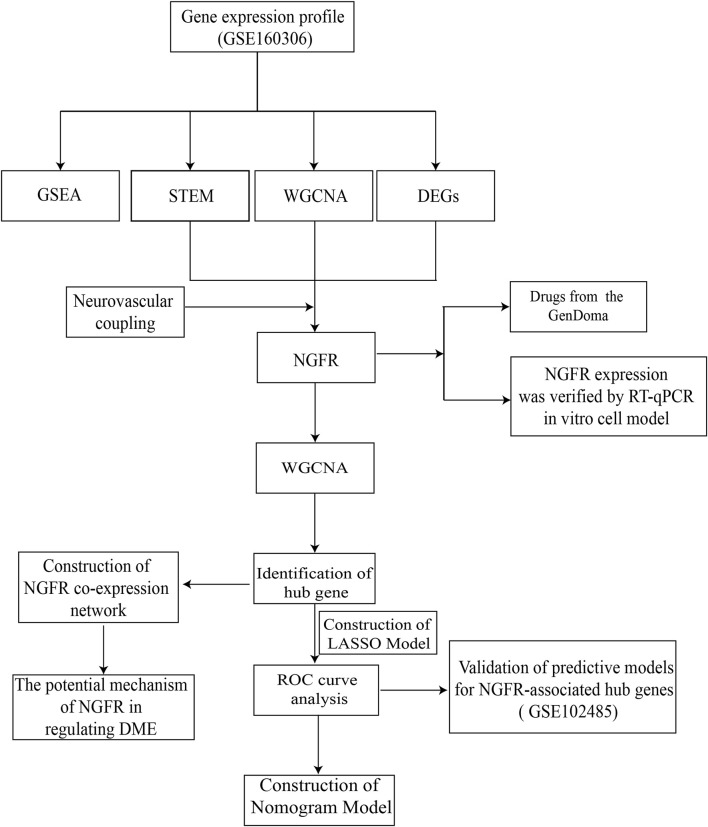
The workflow of gene screening and analyzing.

In this regard, based on abundant public sequencing resources and bioinformatics analysis tools, NGFR-associated with neurovascular coupling and DME was identified by STEM, differential analysis, and WGCNA of GSE160306. The associated biological processes and molecular mechanisms were further investigated by GO, KEGG and GSEA analysis. The ROC curves and nomogram indicated that the model may have comparatively excellent diagnostic performance in the clinical diagnosis of DME. Furthermore, the GenDoma database was searched for potential therapeutic agents targeting NGFR and related hub genes, which could have potential applications for the pharmaceutical development of DME. The workflow was displayed in Figure 1.

## Materials and methods

### Data download and preprocessing

The raw data about human retinal tissue were acquired using GEOquery R package from GSE160306 data set of Gene Expression Omnibus (GEO, https://www.ncbi.nlm.nih.gov/geo/). The high throughput dataset included 79 transcriptomic sample data about diabetic retinopathy. The transcriptomic data from samples of different clinical stages were selected for analysis in this study, including Control (10 samples), Diabetic (10 samples) and DME (8 samples). The highthroughput data was normalized and standardized using Limma R package.

### Weighted gene co-expression network analysis

The gene co-expression network were constructed by the R software analysis toolkit of Weighted Gene Co-expression Network Analysis (WGCNA) (Langfelder and Horvath, 2008). Firstly, abnormal samples were removed through the clustering tree. The correlation coefficients between gene pairs were calculated and the similarity matrix was constructed. A suitable soft threshold power was then chosen to ensure a correlation coefficient threshold of 0.85, and a minimum number of genes in the module was determined to be 100. In order to incorporated possible similar modules, cutting height value 0.25 was defined as the threshold. Hub genes significantly associated with clinical features were identified by two factors, gene significance (GS) and high modular group membership (MM).

### Short time-series expression miner (STEM) analysis

The dataset was analyzed using Short Time-series Expression Miner (STEM) software (version 1.3.13, http://www.cs.cmu.edu/∼jernst/stem) (Ernst and Bar-Joseph, 2006). The processed expression matrix was uploaded. The minimum correlation of the most important parameter used for STEM clustering similar profiles was set to >0.7, with a maximum of 50 model profiles and a maximum unit change between time points of 2. The correlation coefficient determines which model profile each gene was assigned to that was closest to that gene’s expression profile. Standard hypothesis tests were performed under the true time point order of the alignment run to identify which model profiles were associated with appreciably more genes. Clusters were screened according to the significant gene expression trend (*P* < 0.05).

### Identification of differential expression genes (DEGs)

Using the “Limma” package of R software performed DEGs analysis of DME and control samples. We selected genes with a significant difference value of P < 0.05 and abs (log2FC) > 1 as the set of DEGs. The visual plot of differential expression genes was plotted using the “pheatmap” and “ggplot2” packages, respectively.

### Screening of hub neurovascular coupling-related genes

We searched for keywords such as “neurovascular coupling” in GeneCards (https://www.genecards.org/) to screen neurovascular coupling-related genes. In order to screen for hub genes related to neurovascular coupling and DME, the VennDiagram function of Sangerbox was used to intersect important genes obtained from DEGs, WGCNA, STEM and genes from the neurovascular coupling gene set to obtain the final hub genes (Shen et al., 2022).

### Gene set enrichment analysis

Based on the median value of NGFR, GSE160306 was divided into high expression group and low expression group. To further investigate the potential functions of the selected hub gene in DME, gene set enrichment analysis (GSEA) was performed to screen biological process (BP) GO term and KEGG pathways that may be related to DME in GSE160306 datasets. The c5. go.bp.v2023.1. Hs.entrez.gmt and c2. cp.kegg.v2023.1. Hs.entrez.gmt datasets in MsigDB v2023.1. Hs database were chosen as the Reference Gene Set and GSEA analysis was constructed with the R package “ClusterProfiler”. *P* adjusted value (adj. *p* values) < 0.05 was taken as the cutoff criteria.

### Single gene analysis and functional enrichment based on NGFR expression level

Based on the median value of NGFR, GSE160306 was divided into high expression group and low expression group. WGCNA was established according to the expression of NGFR. The abovementioned method was used to set relevant parameters. The modules with the most relevant correlation to NGFR expression were selected to screen related genes, and their biological functions were analyzed by GO and KEGG analysis. Gene Ontology (GO) analysis and Kyoto Encyclopedia of Genes and Genomes (KEGG) were common method for largescale functional enrichment and biological pathways. GO and KEGG enrichment analyses were visualized with the Sangerbox and *P* < 0.05 were considered to be statistically significant. GeneMANIA (http://www.genemania.org) is a resource rich genetic analysis site for constructing gene co-expression network of hub genes (Warde-Farley et al., 2010).

### Establishment of nomogram for predicting DME progression

LASSO regression analysis was performed using the R package glmnet to reduce additional redundant and irrelevant features. The response type of the lasso method was set as binomial, with the DME sample represented when the variables of response were 1 and the control sample represented when the variables of response were 0. In addition, the penalty parameters were adjusted using crossvalidation in order to find the λ with the smallest classification error to determine the variables. After logistic regression analysis, the packaged pROC in R and the receiver operating characteristic (ROC) curve were employed to calculate the area under the curve (AUC), and the diagnostic value of the feature indexes in GSE160306 was analyzed and evaluated. The R package “rms” was used to build the nomogram with characteristic indicators.

### Screening of potential chemical-gene interaction

Drug screening of NGFR was performed by accessing the database of the GenDoma platform (ai.citexs.com).

### Cell culture and treatment

The murine microglia cell line BV-2 was purchased from the Cell Cook (Guangzhou, China). The Muller cells line of rat retina rMC-1 was derived from lixiang eye hospital of soochow university. These cells were cultured in Dulbecco’s Modified Eagle’s Medium DMEM-low glucose (1.0 g/L D-glucose) (Gibco, United States), supplemented with 10% fetal bovine serum (Invitrogen, United States), and maintained at 37°C in a humidified incubator with 5% CO_2_. After cell passage to the fourth generation, the cells were seeded in the six well plate at a density of 1.25 × 10^5^ cells/mL. After approximately 10 h, the cells were incubated with high glucose complete medium (4.5 g/L D-glucose, 10% fetal bovine serum) and sodium palmitate (Kunchuang Biotechnology, Xi’an, China) for 0, 1 h, 3 h, 6 h, 12 h, and 24 h. Then, the cells were collected for further experiments at different time points.

### RNA isolation and real-time quantitative reverse transcription polymerase chain reaction (RT-qPCR)

TRIzol reagent (Beyotime Biotechnology, Shanghai, China) was used to extract total RNA from the specimens, according to the manufacturer’s instructions. Reverse transcription was executed using PrimeScript™ RT reagent Kit (Takara Biomedical Technology, Beijing, China). SYBR Green Master Mix (Beyotime Biotechnology, Shanghai, China) was used to conduct qPCR. Actin was used as an internal reference to standardize the expression data. The primer sequences of mouse NGFR and ACTIN were shown as follows: NGFR_forward: CTAGGGGTGTCCTTTGGAGGT, reverse: CAGGGTTCACACACGGTCT, ACTIN_forward: TGAGCTGCGTTTTACACCCT, reverse: TTTGGGGGATGTTTGCTCCA. The primer sequences of rat NGFR and ACTIN were shown as follows: NGFR_forward: CAGAGGGCACATACTCAGACGA, reverse: ATCTCTTCGCATTCAGCATCAG, ACTIN_forward: ACCCGCGAGTACAACCTTC, reverse: ATGCCGTGTTCAATGGGGTA.

### Analyzing statistics

All bioinformatics analyses were carried out with the software R4.1.1. Nonparametric test or t-test was used to analyze the statistical significance of the differences between the two groups according to the distribution characteristics of the data. *P*-value <0.05 was regarded as statistically significant.

## Results

### Weighted gene co-expression network analysis

**FIGURE 2:**
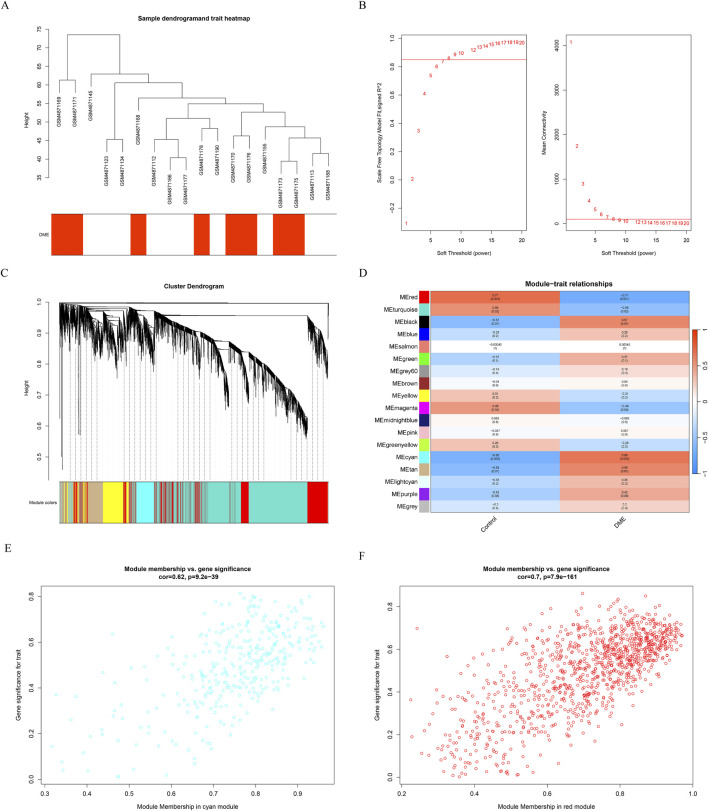
Results of the WGCNA. **(A)** Sample dendrogram and trait heatmap. **(B)** The relevant scale-free topological model fit indices at different soft threshold powers (left). The relevant mean connectivity values at different soft threshold powers (right). **(C)** Cluster dendrogram. **(D)** Correlations between different modules and clinical traits. **(E) (F)** Correlation of module membership (MM) and gene significance (GS) in the cyan and red module.

Gene expression profiles from the diabetes and DME groups were analyzed by clustering according to the clinical characteristics of DME (Figure 2A). A soft threshold of β = 7 (R^2^ = 0.85) was used to make the network scale-free (Figure 2B). The minimum number of genes per gene network module was then set to 100 and the characteristic gene values were calculated for each module, yielding a total of 18 modules (Figure 2C). Cyan and red were the modules most correlated with DME (Figure 2D). The significance of the DME gene in the cyan module was cor = 0.62, P = 9.2e-39 (Figure 2E), and the significance of the DME gene in the red module was cor = 0.7, P = 7.9e-161 (Figure 2F). The 675 genes in these modules associated with DME were retained for further analysis (GS > 0.5, MM > 0.8).

### Identification of time-series genes

**FIGURE 3:**
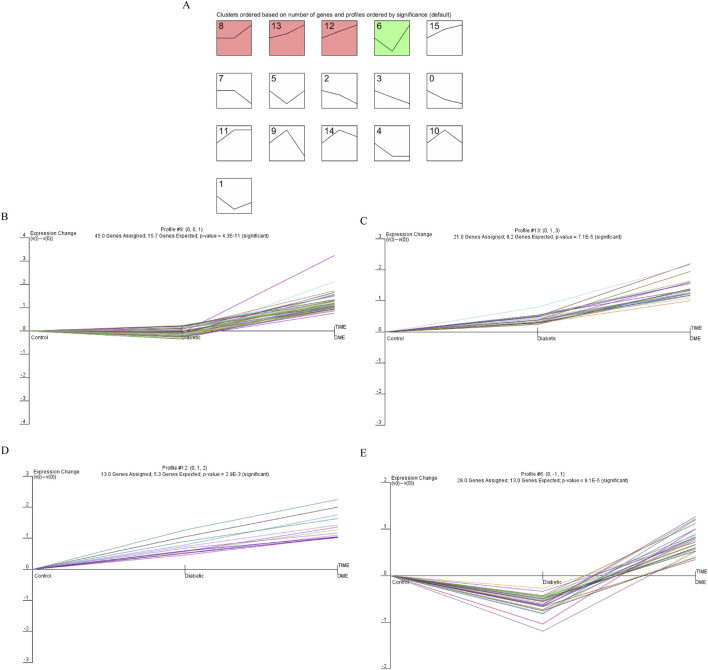
Dynamic expression pattern profiles of diabetic. **(A)** Clustering was identified using short time sequence expression minor clustering analysis. **(B)** Profile 8, **(C)** Profile 13, **(D)** Profile 12, **(E)** Profile 6. Significant clustering of differentially genes in the four models. The x-axis represents the time point. The y-axis is shown at the same scale in the cluster cassette. The different genes clustered in each plot are represented by different colours.

The final gene clusters of interest were identified according to the STEM algorithm with input parameters (c = 2, m = 50), where c indicated the unit of variation and m indicated the number of candidate gene clusters. Figure 3 showed the P-values and fold-changes for the four significant clusters of genes, including profiles 6, 8, 12 and 13. A total of 107 significantly regulated genes were significantly clustered in the four significant clusters as determined by temporal regulation patterns and further STEM analysis. Based on STEM analysis, gene expression was significantly upregulated during the development of diabetes to DME, indicating that these genes were instrumental in the development of DME.

### Identification of hub neurovascular coupling-related gene

**FIGURE 4:**
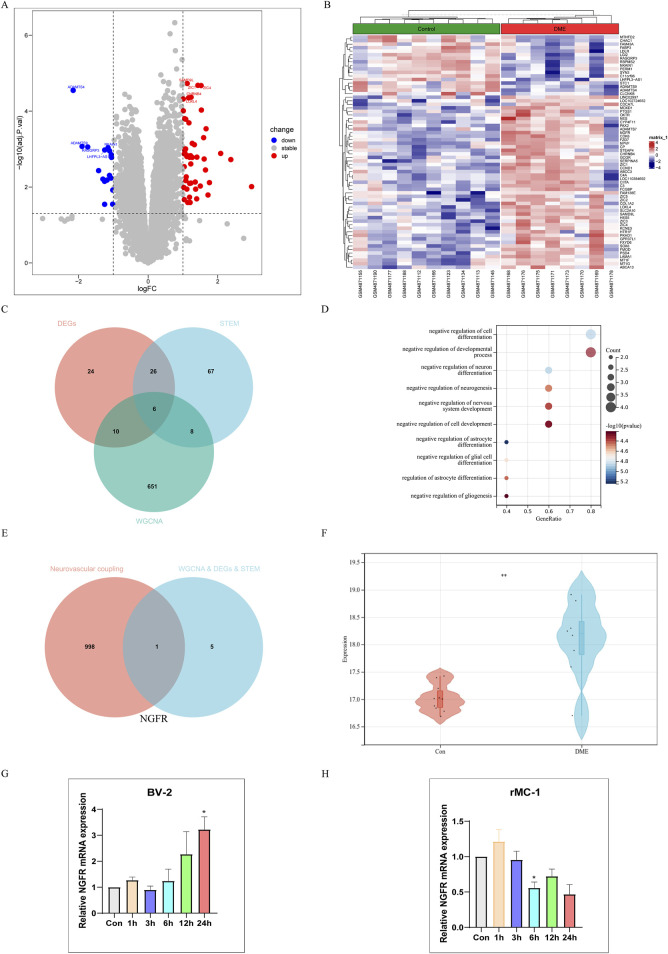
Single gene Analysis Based on NGFR Expression Level. **(A)** Volcano map of difference analysis. **(B)** Heatmap of differential analysis between diabetic and DME group. **(C)** Overlapped genes in differentially expressed genes, DME-related module genes and STEM-related genes (venn diagram). **(D)** Gene Ontology (GO) functional analysis showing enrichment of overlapped genes. **(E)** Overlapped genes between hub genes and neurovascular coupling-related genes (Venn diagram). **(F)** Expression levels of NGFR in Control and DME group (GSE160306). **(G)** The NGFR expression was validated by RT-qPCR in BV-2 (HG and PA (200 μM)). **(H)** The NGFR expression was validated by RT-qPCR in rMC-1 (HG and PA (100 μM)). Values are shown as mean ± SEM from triplicate experiments. Compared with the normal group, *p < 0.05, **p < 0.01, ***p < 0.001, ****p < 0.0001.

*P*-values <0.05, |log2FC| > 1 were obtained as screening conditions for a total of 66 DEGs, which were analyzed differentially by dividing GSE160306 into Control and DME groups. Of these, 17 down and 49 up expressing genes were visualized using volcano plots (Figure 4A) and heat maps (Figure 4B). We intersected 66 DEGs in GSE160306, 107 significantly modulated genes in STEM, and 675 modular genes to obtain 6 genes (Figure 4C). The genes were NGFR, FCGBP, FZD7, GPR37L1, HES5, SLC2A10.

Go analysis found that biological processes of hub genes were mainly related to neuron, including negative regulation of astrocyte differentiation, negative regulation of cell differentiation, negative regulation of neuron differentiation, negative regulation of glial cell differentiation, negative regulation of neurogenesis (Figure 4D). Neurodegeneration occurred earlier than vasculopathy in the process of DME. In order to explore the relationship between neurodegeneration and angiogenesis, we took neurovascular coupling as the key word, hoping to find the key proteins regulating neurovascular coupling. Finally, NGFR was selected as the characteristic gene (Figure 4E). As shown in Figure 4F, the NGFR expression was elevated in DME based on GSE160306. Subsequently, the expression of NGFR was validated by RT-qPCR (Figures 4G, H). In BV-2 cells, the expression of NGFR increased with time, with statistical significance at 24 h. However, in the rMC-1 cell model, the expression of NGFR showed a decreasing trend with the extension of time.

### Verification of biological processes and key pathways by GSEA

**FIGURE 5:**
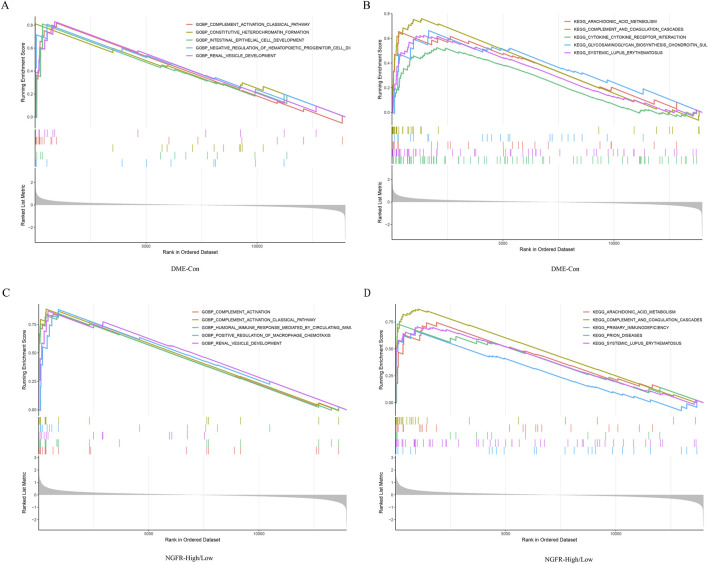
Result of GSEA. **(A)** Biological processes enriched in DME. **(B)** KEGG pathways enriched in DME. **(C)** Biological processes enriched in NGFR-High. **(D)** KEGG pathways enriched in NGFR-High.

Gene set enrichment analysis (GSEA) was performed to explore the potential function. It showed that compared to control samples, biological processes such as positive regulation cell differentiation, positive regulation of tyrosine phosphorylation of stat protein, humoral immune response mediated by circulating immunoglobulin, complement activation, complement activation classical pathway in DME. (Figure 5A). Similarly, compared to NGFR-low, biological processes, such as humoral immune response mediated by circulating immunoglobulin, complement activation, humoral immune response, complement activation classical pathway, β cell mediated immunity were significantly enriched in NGFR-high (Figure 5C). Moreover, the complement and coagulation cascades, cytokine-cytokine receptor interaction, glycosaminoglycan biosynthesis chondroitin sulfate, systemic lupus erythematosus, arachidonic acid metabolism were significantly involved in DME (Figure 5B). Similarly, complement and coagulation cascades, cytokine-cytokine receptor interaction, arachidonic acid metabolism, systemic lupus erythematosus, ecm receptor interaction are also significantly overrepresented in NGFR-high samples (Figure 5D).

### Module associated with NGFR

**FIGURE 6:**
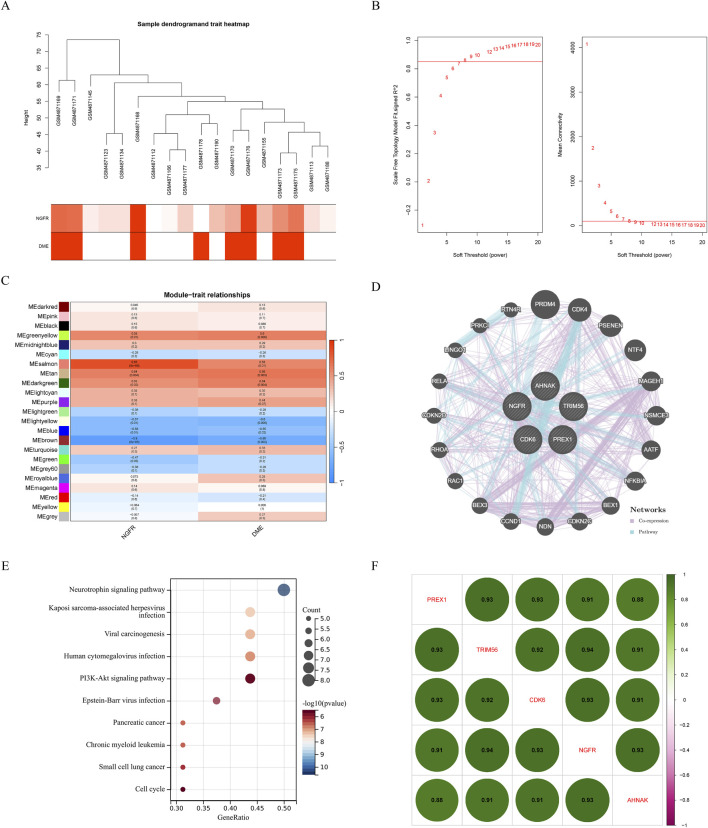
Results of the WGCNA. **(A)** Sample dendrogram and trait heatmap. **(B)** The relevant scale-free topological model fit indices at different soft threshold powers (left). The relevant mean connectivity values at different soft threshold powers (right). **(C)** Correlations between NGFR and different modules. Correlation of MM and GS in the brown, purple and pink module. **(D)** Co-expression network of salmon module hub genes. **(E)** KEGG pathway analysis of salmon module hub genes. **(F)** The correlation of hub genes, amaranth indicates negative correlation and green indicates positive correlation.

To identify the potential regulatory mechanism of NGFR, WGCNA was performed constructing co-expression network based on NGFR expression. The parameters were established by setting the soft-threshold power to 7 (scale free R^2^ = 0.85) (Figures 6A, B). In this study, 16 modules were identified and visualized by heatmap profile (Figure 6C). The association between the modules and NGFR was measured by the correlation between module eigengene (ME) values and NGFR expression. The results showed that the salmon module was the most closely correlated with NGFR (Figure 6C). According to GS > 0.9 and MM > 0.8, 5 genes of salmon were identified as hub genes (NGFR, CDK6, PREX1, TRIM56, AHNAK). In addition, we extract the genes that co-expression with NGFR based on GeneMANIA database (Figure 6D). According to the pathway which these genes are significantly involved in neurotrophin signaling pathway (Figure 6E). Furthermore, the correlation analysis shows that NGFR strongly correlates with hub genes (Figure 6F).

### Establishment of nomogram for predicting DME progression

**FIGURE 7:**
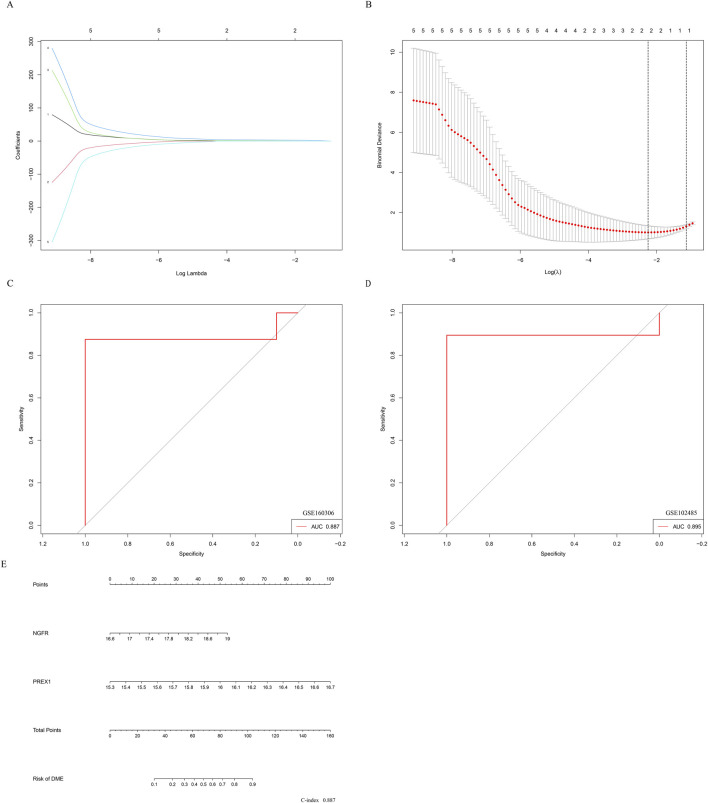
Establishment of nomogram for predicting DME progression. **(A)** The partial likelihood deviance in the jackknife rates analysis. **(B)** The Lasso coefficient distribution plot. Each curve is a candidate feature index. **(C)** The ROC curve was employed to assess the performance of the logistic regression model (GSE160306). **(D)** The ROC curve was employed to assess the performance of the logistic regression model (GSE102485). **(E)** The nomogram was utilized to predicted the occurrence of DME. The characteristic indexes were included in this nomogram.

Therefore, further Lasso regression analysis will hopefully identify candidate indicators that accurately predict DME. In the construction of the LASSO model, the five risk factors of genes were included in the lasso regression (Figure 7A). The LASSO coefficient spectrum for the differentiation index was plotted based on the best λ value of 0.106 (Figure 7B), and a total of 2 potential index, PREX1 and NGFR, were obtained.

Logistic regression was performed on the two characteristic indices and ROC curve was constructed between the DME group and the control group, and its AUC value was 0.887 (>0.7) (Figure 7C). Furthermore, in the GSE102485 dataset, the construction of ROC curves for two characteristic variables was validated, and their AUC was still greater than 0.7 (Figure 7D). As illustrated in Figure 7E, a nomogram was developed to be applied as a therapeutic tool for the diagnosis of DME progression by incorporating characteristic index. In the nomogram, each characteristic index corresponded to a score, and the aggregate score obtained by summing the scores of all feature indices corresponds to the different risks of DME (C-index 0.887). The results showed that the prediction model constructed by the ROC curve and nomogram exhibited good diagnostic performance.

### Screening of potential chemical-gene interaction

**FIGURE 8:**
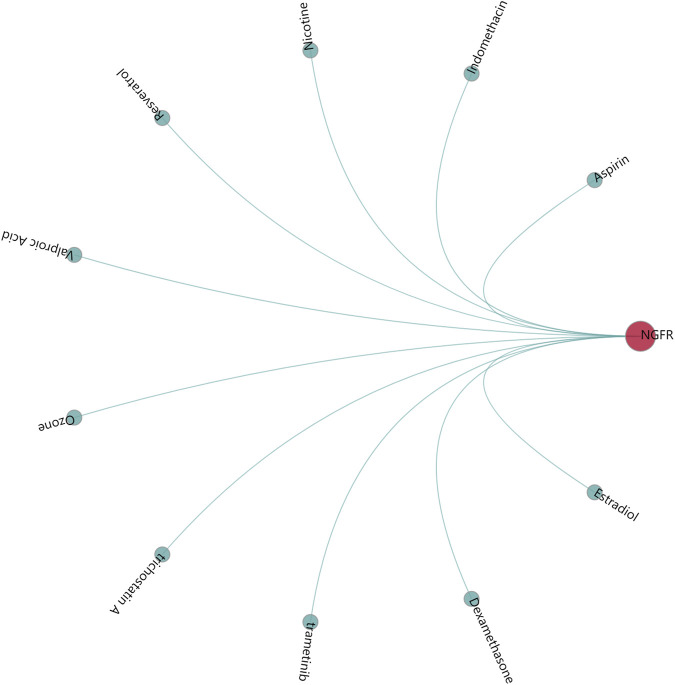
Screening of Potential Chemical-Gene interaction.

GenDoma platform analysis showed that indomethacin, dexamethasone, trametinib, trichostatin A, ozone, valproic acid, resveratrol, nicotine, aspirin were modulators of NGFR, which may regulate the occurrence and development of DME (Figure 8).

## Discussion

Vascular endothelial growth factor is recognized as a critical molecule in the development of macular edema; however, the disease is multifactorial, implicating numerous potential therapeutic targets in its onset and progression (Miller and Fortun, 2018). Existing studies have shown that the development of diabetic macular edema is closely related to the breakdown of the neurovascular coupling mechanism. Neurons and blood vessels are intricately linked within the retinal vascular system, neurovascular coupling is achieved through the coordinated response of various cells in the neurovascular unit (Quiriconi et al., 2024). While the exact molecular mechanisms behind this functional congestion response require further investigation, it is generally accepted that neurovascular coupling damage is an early event in several ocular and systemic diseases, including complications of diabetes (Garhofer et al., 2020; Sheng et al., 2024). Consequently, identifying mediators that regulate neurovascular coupling could unveil new therapeutic targets for the early management of DME.

This study applied WGCNA, STEM and differential gene screening to identify the hub genes in DME. GO enrichment analysis of the identified hub genes revealed that their associated biological processes are primarily related to neuronal functions. Neurovascular coupling is essential for the retinal vascular system to adapt to changes in neural function and metabolic needs (Starace et al., 2021). Finally, our findings indicate that the high expression of NGFR, a gene implicated in neurovascular coupling, may contribute to the development of DME. NGFR, a member of the tumor necrosis factor (TNF) receptor family, can be activated by neurotrophic factors and their precursors through both low and high-affinity binding, respectively (Mirzahosseini et al., 2022).

As a potential new therapeutic target, NGFR plays a crucial role in retinal degenerative diseases such as DR (Galan et al., 2017). Studies in streptozotocin (STZ) mice model of type I diabetic have shown that NGFR mediates the breakdown of neuroglia-vascular units, promotes blood-retinal barrier disruption and neuronal death. Elevated concentrations of NGFR have been detected in the vitreous body of patients with PDR, suggesting that high NGFR expression may be linked to the pathological progression of PDR (Klaassen et al., 2017). In a mouse model of oxygen-induced retinopathy that mimics proliferative DR, NGFR-dependent inflammation leads to ischemia and pathological angiogenesis via Semaphorin 3A (Barcelona et al., 2016). Collectively, these observations indicate that NGFR plays an important role in vascular defects, inflammation and neurodegeneration.

Subsequently, the expression level of NGFR was validated *in vitro* cell model. Experimental evidence from rodent models suggests that neurovascular coupling in the retina is also largely mediated by glial cells (Sorrentino et al., 2016). In the early stage of diabetes, Müller cells and microglia cells have abnormal functions in regulating vascular functions to respond to neural needs (Fletcher et al., 2022). It was verified by RT-qPCR that NGFR was also highly expressed in BV-2 cells stimulated by high glucose and fat. However, in rMC-1 cells, high glucose and fat induction led to decreased expression of NGFR. The reason for this may be that the bioinformatics results are derived from tissues of DME patients and non-diabetic patients, and differences in cell type and culture conditions potentially alter gene expression and provide contradictory results. Therefore, we further explore the regulatory mechanism of NGFR on DME.

To explore the function of NGFR, GSEA enrichment of all genes was performed in GSE160306 to avoid the lopsidedness caused by using only crossgene enrichment. Previous studies have shown that NGFR not only regulates apoptosis signaling but also regulates axon growth, cell cycle, and synaptic plasticity (Bruno et al., 2023). In the STZ-induced mouse model of type 1 diabetes, NGFR mediates the release of ligand-dependent inflammatory cytokines, disrupts glial-vascular units, and promotes blood-retinal barrier breakdown (Barcelona et al., 2016). In our study, functional enrichment analysis by GSEA showed that humoral immune response mediated by circulating immunoglobulin, complement activation, complement activation classical pathway are enriched in DME and NGFR high group. These studies suggest that the high expression of NGFR may not only be involved in the activation of inflammatory response, but also in the immune response to regulate neurovascular coupling, mediating the occurrence and development of DME.

According to the KEGG pathway, the complement and coagulation cascades, systemic lupus erythematosus, cytokine-cytokine receptor interaction, arachidonic acid metabolism were significantly over-represented in DME and NGFR-high samples. Many of these pathways have been previously confirmed to be related to DME. For example, the complement and coagulation cascade has been identified as a significant differential pathway between proliferative DR and normal vitreous (Sun et al., 2021). Our study also found systemic lupus erythematosus, cytokine-cytokine receptor interaction, and arachidonic acid metabolism were associated with DME/NGFR-high samples. These results suggest that the immunity and inflammatory pathway may be involved in regulating the pathogenesis of neurodegenerative diseases, particularly DME.

We also performed WGCNA analysis by characterizing the expression level of NGFR. Among the 16 modules, the salmon module was identified as the key module, with five genes (NGFR, CDK6, PREX1, TRIM56, AHNAK) recognized as hub genes. Further co-expression network construction and functional enrichment showed that NGFR and its expression-related genes were mainly involved in the regulation of neurotrophin signaling pathways. These results suggest that NGFR and its related hub genes may play a significant role in promoting the development of retinal disease through the regulation of neurotrophin signaling pathways. Furthermore, our study found that the high regulation of NGFR expression correlated with the high regulation of CDK6, PREX1, TRIM56, and AHNAK.

In addition, the expression profile of hub genes was extracted to construct LASSO model in GSE160306, where PREX1 and NGFR were identified with high AUC value based on nonzero regression coefficients. Beyond NGFR, the phosphatidylinositol 3,4,5-trisphosphate (PIP3)-dependent Rac exchanger 1 (PREX1) is known to be involved in signal transduction and neuronal migration induced by neurotrophic factors (Ye, 2020). These two genes also obtained better AUC value in ROC curves of GSE102485 data, suggesting their potential for use in diagnosing diabetic complications. Moreover, the nomogram indicated that PREX1 and NGFR were important influencing factors for the occurrence of DME. The results show that the prediction model constructed by NGFR and LRPEX1 may have better diagnostic performance for DME. Finally, several small molecule compounds were identified as potential drug candidates for regulating the hub genes based on research screening, such as indomethacin, dexamethasone, trametinib and trichostatin A.

We identified characteristic DME progression and neurovascular coupling associated genes on the basis of bioinformatics studies. However, there were some limitations in this study. Firstly, bioinformatics studies based on GEO related to neurovascular coupling, which may have a high false positive rate. The mechanism of characteristic genes and the therapeutic effects of these small molecule compounds need to be further elucidated through experiments. Second, in future studies, we will better recruit clinical patients to assess whether NGFR and related gene can be used as a biomarkers and targets for pharmacological intervention in DME.

## Conclusion

In conclusion, this study found that the expression of NGFR and its interacting genes was significantly upregulated in DME. Overexpression of NGFR may affect the immunity and inflammatory pathway, such as complement and coagulation cascades, systemic lupus erythematosus, cytokine-cytokine receptor interaction, arachidonic acid metabolism. Moreover, NGFR may contribute to abnormal neurovascular coupling and be involved in the development of DME by regulating of neurotrophin signaling pathways. NGFR and related genes may be a potential therapeutic target and biomarkers for the control of neurovascular coupling and/or modulation of neurotrophin function in the treatment of DME.

## Data Availability

The original contributions presented in the study are included in the article, further inquiries can be directed to the corresponding authors.
